# Spry1 Is Expressed in Hemangioblasts and Negatively Regulates Primitive Hematopoiesis and Endothelial Cell Function

**DOI:** 10.1371/journal.pone.0018374

**Published:** 2011-04-01

**Authors:** Xuehui Yang, Yan Gong, Robert Friesel

**Affiliations:** Center for Molecular Medicine, Maine Medical Center Research Institute, Scarborough, Maine, United States of America; Wellcome Trust Centre for Stem Cell Research, United Kingdom

## Abstract

**Background:**

Development of the hematopoietic and endothelial lineages derives from a common mesodermal precursor, the Flk1^+^ hemangioblast. However, the signaling pathways that regulate the development of hematopoietic and endothelial cells from this common progenitor cell remains incompletely understood. Using mouse models with a conditional *Spry1* transgene, and a *Spry1* knockout mouse, we investigated the role of Spry1 in the development of the endothelial and hematopoietic lineages during development.

**Methodology/Principal Findings:**

Quantitative RT-PCR analysis demonstrates that Spry1, Spry2, and Spry4 are expressed in Flk1^+^ hemangioblasts *in vivo*, and decline significantly in c-Kit^+^ and CD41^+^ hematopoietic progenitors, while expression is maintained in developing endothelial cells. Tie2-Cre-mediated over-expression of Spry1 results in embryonic lethality. At E9.5 *Spry1;Tie2-Cre* embryos show near normal endothelial cell development and vessel patterning but have reduced hematopoiesis. FACS analysis shows a reduction of primitive hematopoietic progenitors and erythroblastic cells in *Spry1;Tie2-Cre* embryos compared to controls. Colony forming assays confirm the hematopoietic defects in *Spry1;Tie2-Cre* transgenic embryos. Immunostaining shows a significant reduction of CD41 or CD71 and dpERK co-stained cells in *Spry1;Tie2-Cre* embryos compared to controls, whereas the number of VEC^+^ and dpERK co-stained cells is comparable. Compared to controls, *Spry1;Tie2-Cre* embryos also show a decrease in proliferation and an increase in apoptosis. Furthermore, loss of Spry1 results in an increase of CD41^+^ and CD71^+^ cells at E9.5 compared with controls.

**Conclusions/Significance:**

These data indicate that primitive hematopoietic cells derive from Tie2-expressing hemangioblasts and that Spry1 over expression inhibits primitive hematopoietic progenitor and erythroblastic cell development and expansion while having no obvious effect on endothelial cell development.

## Introduction

Primitive hematopoietic cells (HCs) arise in the yolk sac from mesoderm-derived cells called blood islands (Bls) [1]. The possibility of a common progenitor for endothelial cells (ECs) and HCs, termed the hemangioblast, has been proposed based on the observation that ECs and HCs emerge from BIs in proximity and at a similar time during embryonic development. Studies in embryonic stem (ES) cells indicate that blast colony-forming cells (BL-CFU) lead to both HCs and ECs in vitro [2], [3]. An alternative to this bi-potential common precursor theory shows the first hematopoietic cells emerging from phenotypically differentiated endothelial cells that have hematopoietic potential (i.e. hemogenic endothelium) [4]. Fate mapping reveals that hematopoietic cells originate from VE-Cadherin (VEC) positive endothelial cells [5], suggesting that a subset of definitive hematopoietic cells originate directly from hemogenic endothelial cells. Recently, in vivo time-lapse imaging of the dorsal aortic floor of mouse and zebrafish provide direct evidence that hematopoietic cells emerge from aortic endothelium [6], [7], [8]. Furthermore, the hemangioblast generates hematopoietic cells through a hemogenic endothelium stage and thus provides a link between these two hypotheses [9].

The control of the formation of the hemangioblast and subsequent formation of hematopoietic and endothelial cells from a common progenitor remains unclear. Many growth factors and cytokines regulate hemangioblast formation, and subsequent hematopoietic and angiogenic differentiation [10]. Studies on embryonic stem cells show that fibroblast growth factor-2 (FGF2) and activin A induce the differentiation of mesodermal precursors to a hemangioblastic fate. However, the role of FGF and fibroblast growth factor receptor (FGFR) signaling on hematopoietic and endothelial cell differentiation is still controversial. Loss of FGFR1 function studies in murine embryonic stem cells showed that FGFR1 signaling is required for hematopoietic but not endothelial cell development [11]. In contrast, in the chick, high FGF activity inhibits primitive hematopoiesis and promotes an endothelial cell fate, whereas inhibition of FGFR activity leads to ectopic blood formation and down-regulation of endothelial markers [12].

Flk1 (VEGFR2), one of the receptors for vascular endothelial cell growth factor (VEGF), is a marker for lateral plate mesodermal and the earliest differentiation marker for endothelial and hematopoietic cells. VEGF/Flk1 signaling mediates proliferation, migration, and differentiation. Disruption of *Flk1* results in embryonic lethality between E8.5 to E9.5 with an absence of blood islands at E7.5 and no organized blood vessels in vivo [13]. However, *Flk1−/−* ES cells can differentiate into both lineages in vitro [14], indicating that Flk-1 is required for the migration of progenitors into the proper microenvironment during embryogenesis. In addition, VEGF is also required for the production of fully committed hematopoietic progenitors. Heterozygous inactivation of the *VEGF* gene results in impaired development of the vascular and hematopoietic systems [15], [16]. In the chicken, a high concentration of VEGF inhibits the differentiation of hematopoietic progenitor cells (HPCs) from VEGFR2^+^ cells [17]. These data indicate that precise regulation of FGFR and VEGFR signaling is necessary for proper hemangioblast formation, migration and subsequent hematopoietic and endothelial development.

Sproutys (Sprys) were identified as feedback regulators that restrain receptor tyrosine kinase (RTK) signaling intensity and duration [18], [19]. Over-expression of Spry4 by adenoviral infection of mouse embryos inhibited angiogenesis in vivo [20]. Compound knockout of the *Spry2* and *Spry4* genes in mice leads to cardiovascular and other defects and *Spry4−/−* mice have accelerated angiogenesis in response to injury [21]. Morpholino oligonucleotide mediated knock down of Spry4 in zebrafish leads to hematopoietic defects [22]. However, the roles of Sprys in early endothelial development and hematopoiesis have not been addressed in mammals. In the present study, we found that Sprys are expressed in Flk1^+^ hemangioblasts and continually expressed in developing endothelial cells, however expression is decreased in hematopoietic c-Kit^+^ and CD41^+^ cells. Because Tie2 is expressed in Flk1^+^ hemangioblasts, beginning at E7.5, we used Tie2-Cre to generate conditional Spry1 transgenic mice in this study. Over-expression of Spry1 in Tie2-Cre expressing cells results in embryonic lethality between E10.5 to E11. Further characterization of *Spry1;Tie2-Cre* transgenic embryos showed a severe reduction in primitive hematopoietic progenitor and erythroblastic cells, but had normal endothelial cell formation at E9.5. In contrast, loss of Spry1 leads to an increase in CD71^+^ progenitor cells at E9.5, although this is not a fully penetrant phenotype. Furthermore, over-expression of Spry1 increases apoptosis and decreases cell proliferation and is associated with decreased pERK in CD41^+^cells. Together, our results indicate a decrease of Spry1 expression during hemangioblast committing to hematopoietic progenitors is necessary for hematopoietic cell development and expansion, whereas endothelial cell development is relatively unaffected.

## Results

### The expression of Sprys changes with the stage of hematopoietic cell differentiation

**Figure 1:**
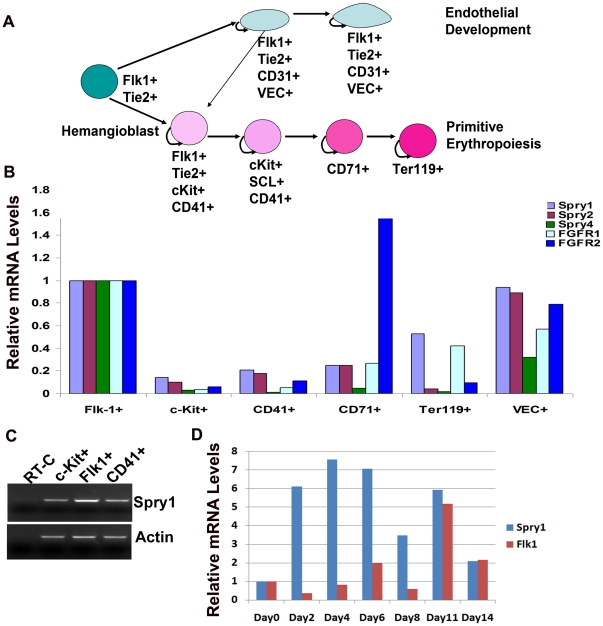
Quantitative expression of Spry1, Spry2, and Spry4 during hematopoietic and endothelial cell development. (A) Schematic illustration of endothelial and hematopoietic cell development. (B) RT-qPCR from sorted cells to show stage-dependent Sprys expression in hematopoietic cell population and relative steady-state expression in endothelial cells. (C) RT-PCR to confirm the expression pattern of Spry1 in hematopoietic cell development. (D) RT-qPCR of mRNA from differentiating embroid bodies to show the Spry1 expression pattern during ES cell differentiation.

Although Spry genes are expressed developmentally in a variety of mouse tissues, endogenous expression in the hematopoietic and endothelial cell precursors has not been characterized. Flk1^+^ mesodermal cells give rise to the hemangioblast in early embryogenesis. Figure 1A outlines the commitment and differentiation of primitive hematopoietic and endothelial cells, in which Flk1^+^ mesoderm cells give rise to c-Kit^+^, CD41^+^ primitive hematopoietic progenitors and also CD31^+^, VEC^+^ endothelial cells. A small fraction of the Flk1^+^, CD31^+^, VEC^+^ hemogenic endothelial cells can also differentiate into hematopoietic cells [6], [7], [8]. Because hematopoietic progenitor cells express Tie2, Flk1, and CD31 endothelial markers and c-Kit and CD41 hematopoietic markers, we used fluorescence activated cell sorting of E9.5 embryonic cells, and isolated Flk1 single positive cells as the hemangioblast population, VEC^+^ cells as endothelial cells, cKit^+^, CD41^+^ as primitive hematopoietic progenitors, CD71^+^ and Ter119^+^ cells as erythroblastic cells. Wild type E9.5 embryo or yolk sac cells were pooled, and aliquots triple stained with fluorescent antibodies Flk1-PE, c-Kit-APC and CD41-FlTC or CD71-APC, Ter119-PE, and VEC-FITC and sorted. RT-qPCR of sorted cells showed a biphasic expression pattern of Spry1, which is expressed in Flk1^+^ progenitor cells, but declines in c-Kit^+^ and CD41^+^ cells, and increases again in Ter119^+^ cells (Figure 1A, B). Spry2 is expressed in Flk1^+^ progenitor cells, and Spry2 expression parallels that of Spry1 until the Ter119^+^ stage where its expression is down regulated. Spry4 is expressed in Flk1^+^ progenitors, but is down regulated during subsequent stages of hematopoietic development. In contrast, Spry1 and Spry2 expression levels in VEC^+^ are similar to Flk-1^+^ cells, but Spry4 expression levels are lower. We also observed a strong increase in the expression of FGFR2 in CD71^+^ cells, which subsequently declined in Ter119^+^ cells. This correlates well with previous observations that indicate that FGFR2 plays a role in hematopoiesis [12], [23]. *In vitro* differentiation of mouse ES cells using the hanging drop method without LIF showed induction of Spry1 expression is biphasic, with the first peak of expression occurring on day 4 of differentiation, before the rise in Flk1 expression (Figure 1C). A second peak of Spry1 expression occurs around day 11 when Flk1 expression has peaked. Spry4 expression was relatively unchanged during ES cell differentiation (data not shown).

### Over expression of Spry1 in Tie2 expressing cells is embryonic lethal

**Figure 2:**
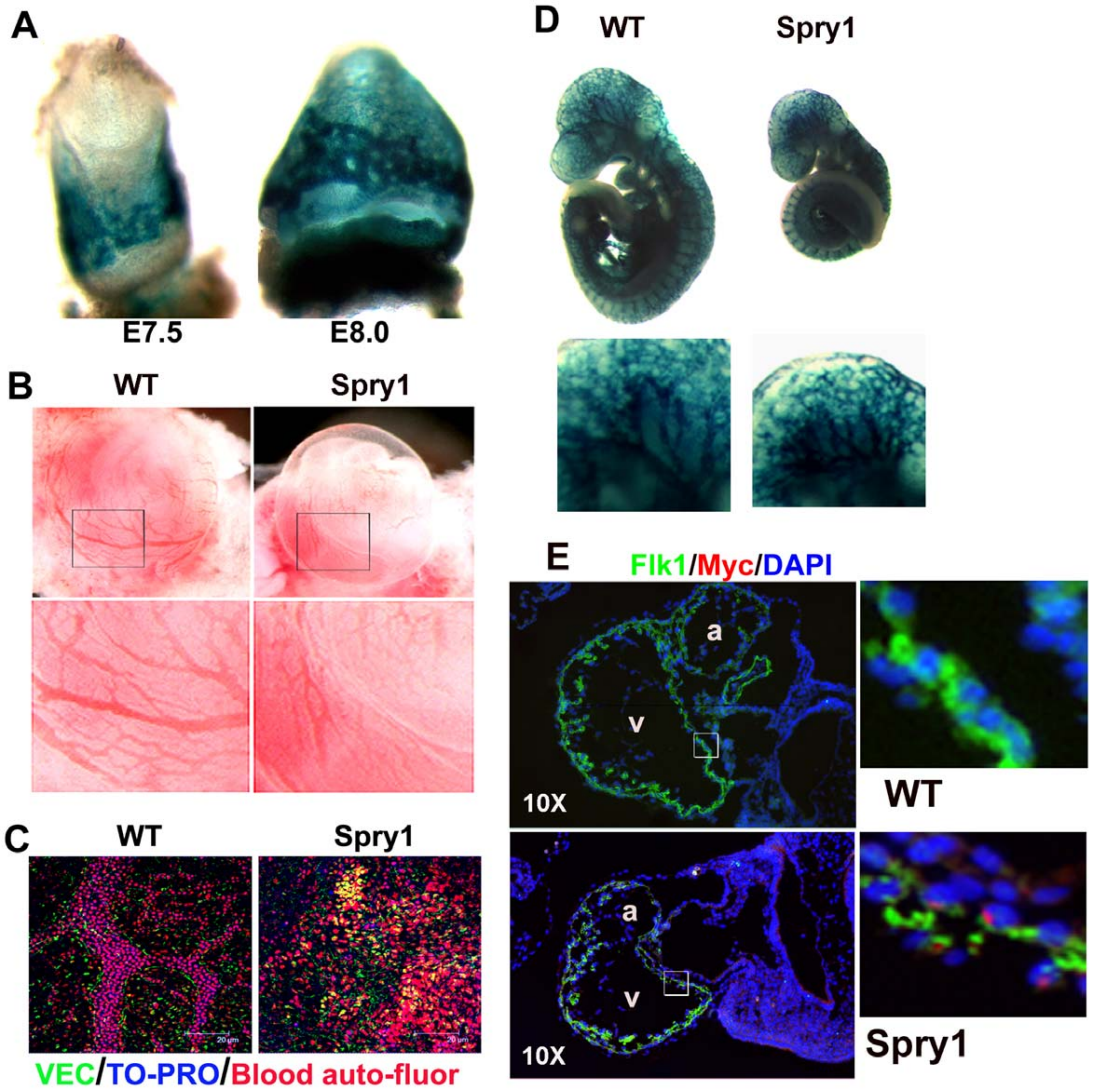
*Spry1;Tie2-Cre* transgenic embryos exhibited an anemic phenotype and vascular defects. (A) The Rosa26LacZ reporter shows efficient Tie2-Cre recombination and the pattern of transgene expression. (B) *Spry1;Tie2-Cre* (Spry1) transgenic embryos showed an anemic phenotype, and a lack of large vessel development in the yolk sac compared to littermate controls (WT). (C) Whole mount immunostaining of Spry1:Tie2-Cre (Spry1) yolk sacs at E9.5 with VEC antibodies shows a reduction in the formation of larger vessels compared to wild type controls (WT). The unmerged images are shown in Supplemental figure 2. (D) Whole mount X-gal staining showed a relative normal vascular pattern in *Spry1;Tie2-Cre* embryos compared to controls. (E) Flk1 and Myc co-immunostaining shows transgene expression of Spry1 in Flk1^+^ cells, but reveals a discontinued endocardium in *Spry1;Tie2-Cre* embryos compared to controls.

The Tie2 gene encodes an angiopoietin receptor, its expression is detected as the first endothelial cell arise, and remains positive in endothelial cells throughout development, and in all adult endothelial cells [24], [25]. Tie2 is not only expressed in adult and embryonic ECs but in extra-embryonic Flk1^+^ cells during gastrulation, and in hematopoietic progenitor cells [26]. *Tie2-Cre* transgenic mice have been extensively characterized [27], [28] and exhibit a similar expression pattern to *Tie2-LacZ* transgenic [29]. In addition, it was recently shown that the recombination efficiency induced by *Tie2-Cre* transgenic mice was ∼85% using Rosa26R-EYFP and FACS analysis as the readout [30]. We confirmed that Flk1^+^ positive cells also express Tie2 in E7.5 and E9.5 embryos by FACS (data not shown). To evaluate the role of Spry1 in the development of hematopoietic and endothelial cell lineages, we conditionally over-expressed Spry1 in Tie2-expressing hemangioblastic cells using a Cre/LoxP strategy, which has been described in detail elsewhere [31]. Crossing Tie2-Cre mice with CAGGFP-Spry1 mice activated Spry1 expression; embryos that genotype positive for both transgenes are called *Spry1;Tie2-Cre*. Tie2-Cre mediated recombination was confirmed in extra embryonic blood islands of E7.5 *Spry1;Tie2-Cre;Rosa26LacZ* embryos by β-galactosidase staining (Figure 2A and data not shown). This staining spread to all vascular components of the yolk sac and embryo by E8.0. *Spry1;Tie2-Cre* transgenic embryonic yolk sacs show a 2–8-fold increase in expression of Spry1 mRNA by qPCR (Figure S1). Over-expression of Spry1 in Tie2-Cre expressing cells resulted in an anemic phenotype (Figure 2B) and embryos died between E10.5 and E11. Immunostaining of E9.5 yolk sacs with VEC antibodies showed a disruption in vascular integrity and the formation of larger vessels in *Spry1:Tie2-Cre* embryos but not in controls (Figure 2C, and l Figure S2). Autofluorescent blood cells were scattered throughout the yolk sac of *Spry1;Tie2-Cre* embryos, but were contained within larger vessels in wild type control yolk sacs. We observed an apparent increase in VEC^+^ cells in *Spry1;Tie2-Cre* yolk sacs compared to controls, although these cells were not organized into larger vessels suggesting a defect in vascular remodeling or may reflect hemangioblastic cells that are in transition to hematopoietic cells but express residual VEC (Figure S2). β-galactosidase and isolectin Grifonia simplicifolia (IB4) staining shows that at E9.5 the vasculature has formed in *Spry1;Tie2-Cre* embryos, but the embryos are smaller compared to controls (Figure 2D, and data not shown). The reduced size of *Spry1;Tie2-Cre* embryos is likely a secondary effect due to defects in hematopoiesis and anemia. Co-immunostaining with Flk1 and Myc antibodies showed that the *Spry1* transgene was expressed albeit incompletely in Flk1^+^ endocardial cells lining the heart (Figure 2E). This immunostaining also revealed that *Spry1:Tie2-Cre* embryos had a discontinuous Flk1^+^ endocardium, whereas control embryos showed a continuous Flk1^+^ endocardium (Figure 2E).

**Figure 3:**
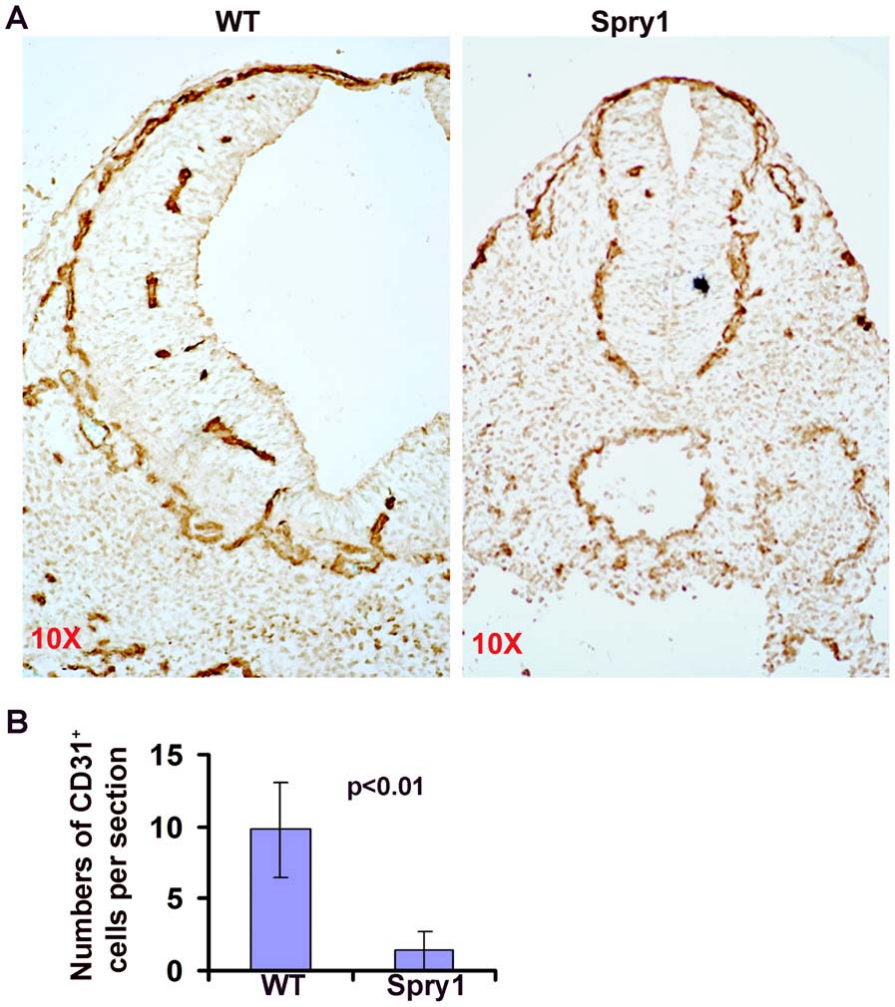
Over-expression of Spry1 inhibits vascular invasion of the neural tube. (A) E9.5 embryo transverse sections were stained with anti-CD31 antibodies followed by HRP-conjugated anti-rabbit antibody. The results showed a normal vascular plexus surrounding the neural tube in both *Spry1;Tie2-Cre* (Spry1) and control embryos (WT), however the vascular plexus invaded the neural tube in wild type but not in *Spry1;Tie2-Cre* transgenic embryos. (B) Quantification of CD31^+^ cells in the neural tube of E10 embryos. CD31^+^ cells were counted in the neural tubes of five sections from at least 3 wild type and *Spry1;Tie2-Cre* embryos.

Although β-galactosidase staining of E9.5 *Spry1;Tie2-Cre* embryos showed apparently normal vasculature (Figure 2D), sections through the heart showed a discontinuous endocardium (Figure 2E). To determine whether other vascular defects were present in *Spry1;Tie2-Cre* embryos, we stained transverse sections of E9.5 embryos with Flk1, VEC and PECAM antibodies. Immunofluorescent staining showed CD31^+^, Flk1^+^ and VEC^+^ cells invading the neural tube of control embryos (Figure 3, supplemental Figure S3 and data not shown). In contrast, the neural tube of *Spry1;Tie2-Cre* embryos was devoid of CD31^+^ , Flk1^+^ and VEC^+^ cells indicating a defect in vascular invasion of the neural tube. These results suggest that endothelial cells form in the presence of over expressed Spry1 but that there are defects in vascular remodeling or secondary developmental defects likely due to the anemia phenotype.

### Over expression of Spry1 in Tie2-expressing cells impairs primitive hematopoiesis but not endothelial cell development

To further characterize the anemic phenotype of *Spry1;Tie2-Cre* transgenic embryos, we analyzed the expression of hematopoietic and endothelial marker genes by quantitative RT-PCR of E9.5 embryos or yolk sacs. The expression of genes associated with hematopoietic development including Gata1, Runx1, and beta-like embryonic hemoglobin chain 1 (ßH1) were significantly down regulated in *Spry1;Tie2-Cre* yolk sacs and embryos compared to controls. We also observed a down-regulation of Tie1, Tie2, Flt1, Flk1, and VEC, but very little difference in the expression of mesodermal marker Brachyury (Figure S4A and data not shown).

**Figure 4:**
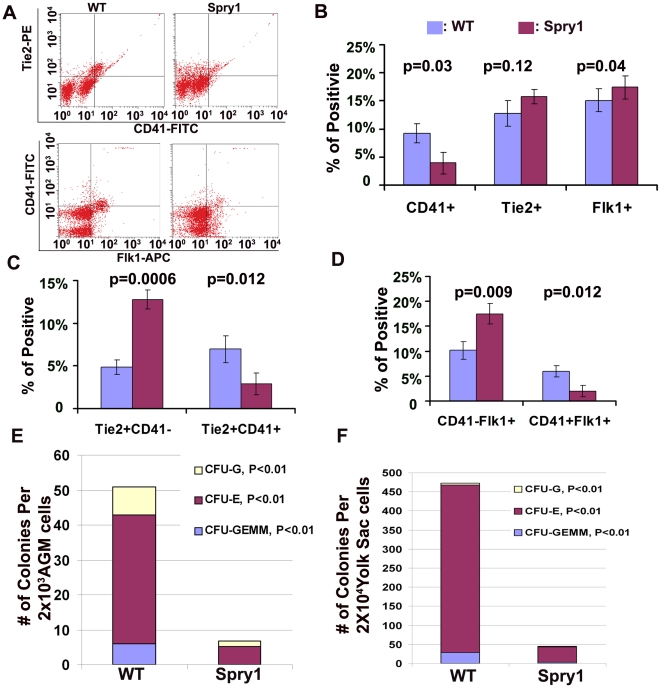
Over-expression of Spry1 in Tie2-expressing cells impairs formation of hematopoietic cells. (A) Representative FACS analysis of E9.5 yolk sacs. (B) Quantification of FACS analysis on E9.5 yolk sacs show that over-expression of Spry1 significantly decreased CD41^+^ primitive hematopoietic progenitor, while the numbers of total Tie2^+^ or Flk1^+^ cells are not significantly affected. (C, D) Quantification of FACS analysis on E9.5 yolk sacs showed that over-expression of Spry1 significantly decreased Tie2^+^,CD41^+^ or Flk1^+^,CD41^+^ newly emerged hematopoietic progenitors, but increased Tie2^+^,CD41^−^ or Flk1^+^,CD41^−^ hemangioblast and/or endothelial cells. (E, F) Colony forming assay on Methocult GF medium showed that *Spry1;Tie2-Cre* transgenic embryo AGMs and yolk sacs formed far fewer hematopoietic colonies compared to controls.

Although Tie2, Flk1 and VEC are markers of endothelial cells, they are also expressed in hemangioblasts and transiently expressed in intermediate progenitors during early hematopoietic cell development [26], [32]. To evaluate the defect in *Spry1;Tie2-Cre* embryos at the cellular level, fluorescence-activated cell sorting (FACS) was performed on E9.5 embryo and yolk sac cells and shows that Tie2^+^,CD41^+^ or Flk1^+^,CD41^+^ hematopoietic progenitors, CD71^+^ erythroblasts, and Ter119^+^ erythroid cells are significantly reduced in *Spry1;Tie2-Cre* embryos compared with controls. However, the number of Tie2^+^, CD41^−^ and Flk1^+^,CD41^−^ endothelial progenitor cells are increased relative to the hematopoietic progenitor cells, although the total numbers of Tie2^+^,Flk1^+^ or the numbers of Tie2^+^,Flk1^+^ cells are similar between *Spry1;Tie2-Cre* embryos and controls (Figure 4A, B, C, D, Figure S4 and data not shown). Because Tie2 expression begins at E7.0 [26], Flk1^+^ hemangioblastic cells, which also expressing Tie2, lead to both hematopoietic and endothelial cells and are derived from the mesoderm beginning at E7.5, we therefore examined whether over-expression of Spry1 affects early Flk1^+^ cell differentiation. FACS analysis of E8.5 embryos showed the number of Flk1^+^ cells is similar between *Spry1;Tie2-Cre* embryos and controls, however the number of CD41^+^ cells is decreased in *Spry1;Tie2Cre* embryos compared to controls (Figure S4F). Thus, forced expression of Spry1 in Flk1^+^ cells leads to a defect in the commitment of these cells to the CD41^+^ lineage.

To further evaluate the hematopoietic defects due to over-expression of Spry1, we performed in vitro methylcellulose colony forming assays on E9.5 AGMs or yolk sacs. Over expression of Spry1 resulted in fewer erythroid and myeloid colonies compared to controls (Figure 4E, F). Together these data suggest that forced expression of Spry1 in Tie2-expressing cells impairs primitive hematopoietic differentiation, but not endothelial cell development.

### Over expression of Spry1 in Tie2-expressing cells results in a decrease in cell proliferation and increased apoptosis in part through inhibition of ERK phosphorylation

**Figure 5:**
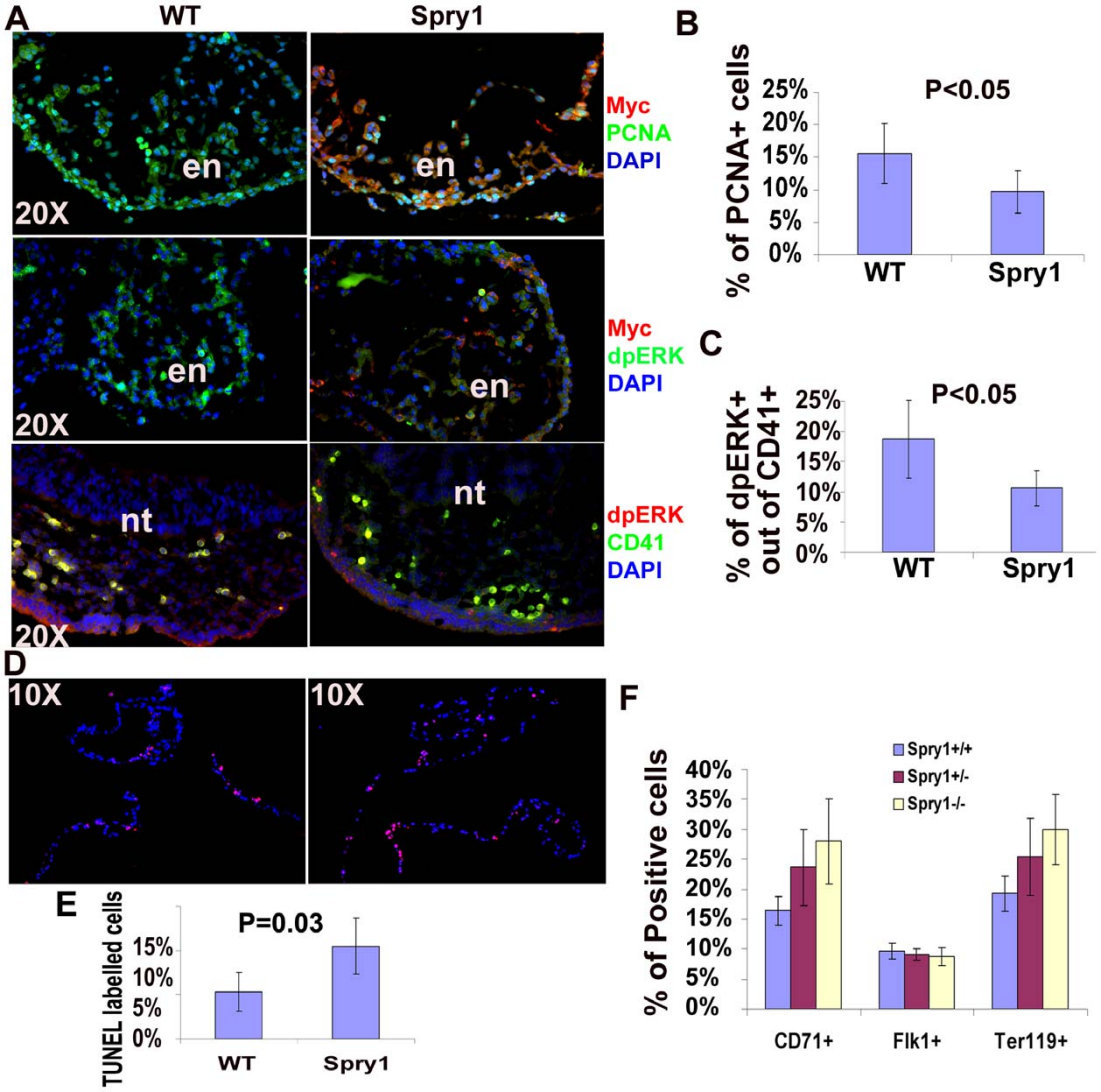
Forced expression of Spry1 impairs cell proliferation and survival in part through inhibiting ERK activation whereas depletion of Spry1 leads to an increase in hematopoietic cell formation at E9.5. (A) Representative images of PCNA, dpERK, CD41 and Myc immunofluorescence staining on E9.5 embryo sections (original magnification of 100×). (B) Quantification of percentage of PCNA^+^ cells normalized by total DAPI cells. (C) Quantification of percentage of dpERK^+^ cells normalized by CD41^+^ cells. (D) TUNEL staining of E9.5 yolk sac sections showed an increase in TUNEL positive cells. (E) Quantification of TUNEL staining of D normalized to total DAPI^+^ cells. (F) Quantification of FACS analysis on E9.5 yolk sacs showed an increase of CD71^+^ and Ter119^+^ cells in *Spry1−/−* and *Spry1+/−* embryos compared to *Spry1+/+* control embryos.

One of the functions of Spry proteins is to inhibit RTK-mediated ERK activation and subsequent cell proliferation and survival *in vitro*. Therefore, it was of interest to determine whether forced expression of Spry1 in Tie2 expressing cells *in vivo* had a similar effect. Co-immunofluorescence staining was employed on frozen sections of E9.5 embryos, and showed a significant reduction of CD41^+^ or CD71^+^ and dpERK co-stained cells in *Spry1;Tie2-Cre* embryos compared to controls (Figure 5A, C and data not shown). Interestingly, we observed that most of the intravascular CD41^+^ and CD71^+^ cells were not stained with dpERK both in *Spry1;Tie2-Cre* or control embryos, and there were only few VEC^+^ stained cells that were dpERK positive (data not shown). PCNA staining showed a reduction in proliferating cells in *Spry1;Tie2-Cre* embryos compared to control (Figure 5A, B). We also noted that there were few CD41^+^ or CD71+ and Myc-Spry1 co-stained cells, but most of VEC^+^ stained cells were Myc-Spry1 positive in *Spry1;Tie2-Cre* embryos (data not shown). TUNEL labeling showed a 2-fold increase in apoptosis in *Spry1;Tie2-Cre* yolk sacs compared to control (Figure 5D, E). Together these data indicate that over-expression of Spry1 in Tie2 expressing cells in vivo inhibits hematopoietic cell proliferation and survival, and increases apoptosis in part through inhibition of ERK activation. While other pathways such as Akt may be involved, we could not detect changes in Akt signaling by immunohistochemistry (data not shown).

### Loss of Spry1 results in an increase in CD71^+^ and Ter119^+^ cell populations

The hematopoietic defects in *Spry1;Tie2-Cre* transgenic mice prompted us to evaluate whether loss of Spry1 function will increase hematopoietic cell formation during embryogenesis. Therefore, we analyzed hematopoietic cell populations from E9.5 *Spry1−/−* embryos using FACS analysis. The results show a dose-dependent increase of CD71^+^ and Ter119^+^ hematopoietic cells in *Spry1+/−* and *Spry1−/−* compared to *Spry1+/+* E9.5 embryos (Figure 5F). The phenotypic penetrance is about 40%, whereas the Flk1^+^ cell population is comparable between *Spry1+/+*, *Spry1+/−* and *Spry1−/−* embryos. Therefore, down-regulation of Spry1 favors the development and expansion of primitive hematopoietic cells from hemangioblast precursors. Thus, normal primitive hematopoietic development is sensitive to Spry1 levels.

## Discussion

Spry inhibition of RTK-mediated ERK activation and subsequent inhibition of cell proliferation, migration, and differentiation has been documented in several cell types *in vivo* and *in vitro* [33], [34], [35], [36]. Morpholino oligonucleotide-mediated knock down of Spry4 in zebrafish leads to hematopoietic defects, however the mechanism is unclear [22]. The role of Sprys in endothelial and hematopoietic development during mammalian embryogenesis has not been described. Here, we demonstrate that Spry1, Spry2, and Spry4 are expressed in Flk1^+^ mesodermal cells and their expression is maintained in VEC^+^ endothelial cells, but declines in c-Kit^+^, CD41^+^ hematopoietic cell lineages, suggesting that Sprys may regulate the differentiation of the hematopoietic and endothelial cell lineages differently. Indeed, *Spry1;Tie2-Cre* transgenic embryos displayed significant reductions in CD41^+^, CD71^+^ and Ter119^+^ hematopoietic populations, whereas loss of Spry1 results in increased CD41^+^, CD71^+^ and Ter119^+^ hematopoietic populations in a gene dosage-dependent manner. Furthermore, we show that over-expression of Spry1 decreases CD41^+^ or CD71^+^ cell proliferation and survival that correlated with a decrease in ERK activation in these cell populations. However, endothelial cell development and proliferation was relatively unaffected in this context. Thus, our findings are of importance not only for demonstrating the ERK signaling pathway is important to hematopoietic cell expansion and survival, but also for a better understanding of the role of Sprys in differentiation and the subsequent expansion of hemangioblasts that lead to the hematopoietic and endothelial lineages.

Hematopoietic differentiation and subsequent proliferation from mesodermal stem cells are critical to the generation and maintenance hematopoietic cell populations. Cytokines and growth factors, such as FGF, VEGF-A, angiopoietin, c-Kit ligand, BMPs and interleukins, have been shown to be important in maintaining hematopoietic stem cell expansion and hematopoiesis *in vitro* and *in vivo* [10], although the specific role of each signal pathway remains unclear. Hematopoietic cytokines and growth factors mediate cell proliferation in part through the ERK pathway. ERK activation mediates proliferative effects through downstream transcription factors including NF-κB, Ets-1, CREB, AP-1, c-Myc and others. These transcription factors induce expression of genes important for cell-cycle progression, such as cyclins and CDKs, and Bcl-2, which promotes cell survival. Mice lacking Mek1 display a reduction in CD4^+^/CD8^+^ thymocytes due to a defective proliferation response of the T-cell receptor [37]. Loss of Gab2, an adaptor protein involved in PI3K and ERK signal pathways, leads to defects in multi-lineage hematopoietic cell expansion [38]. In this study, we demonstrate that a proliferative hematopoietic defect in *Spry1;Tie2-Cre* transgenic embryos is associated with significant decreases of CD41^+^ or CD71^+^ and dpERK double positive cells, suggesting that ERK activation is important for hematopoietic expansion during embryogenesis.

Contradictory roles for FGFR signaling in the regulation of hematopoiesis have been reported, with FGFR1 having a positive effect, whereas FGFR2 negatively regulates hematopoiesis in mouse and chick embryogenesis, respectively [11], [12], [23]. We have shown a stage-dependent expression pattern of FGFR1 and FGFR2 during hemangioblast differentiation into primitive hematopoietic cells. Both FGFR1 and FGFR2 are highly expressed in Flk1^+^ hemangioblasts, and decline in cKit^+^, CD41^+^ primitive hematopoietic progenitors. Subsequently FGFR2 gradually increases during further differentiation of hematopoietic cells, while the peak expression of FGFR1 is in CD71^+^ cells but decreases in more differentiated Ter119^+^ cells. This expression pattern correlates well with the expression of Sprys, in agreement with the concept that FGF/FGFR signaling regulates Sprys expression. Our results suggest that: 1) FGF/FGFR signaling may play a role in mesodermal Flk1^+^ cell formation and expansion, 2) down-regulation of FGF/FGFR signaling may favor the commitment of Flk1^+^ to the hematopoietic lineage, 3) FGFR1 may promote the expansion of CD71^+^ erythroblasts but may not be required for further differentiation and maturation, and 4) FGFR2 may positively regulate erythrocyte differentiation and maturation. Our results also suggest that the feedback circuit between FGFR signaling and Sprys may be necessary for the hematopoietic homeostasis. Further study is required for a better understanding the role of FGF/FGFR signaling in the regulation of primitive hematopoiesis.

The Tie2 receptor is expressed in mature endothelial cells, endocardium and in the hemangioblast, a common precursor that gives rise to hematopoietic and endothelial lineages. FACS analysis of pooled normal E8.5 embryo and yolk sac cells showed about 10.3% of Tie2^+^ cells co-expressing c-Kit, and 2.3% of Tie2^+^ cells co-expressing CD41 (Figure S5) confirming this concept. However, the Myc-tagged Spry1 transgene in *Spry1;Tie2-Cre* embryos was mainly detected in endothelial and endocardial cells, and only a few CD41^+^ cells had detectable Myc-tagged Spry1 transgene. *Rosa26LacZ* reporter staining indicated that Tie2-Cre mediates efficient recombination in our transgenic model. Therefore, it is conceivable that over-expression of Spry1 impairs the survival or viability of CD41^+^ and CD71^+^ cells. Indeed, a significant increase in apoptosis occurred in hematopoietic cells of *Spry1;Tie2-Cre* mice compared to controls.

Forced expression of Spry4 in endothelium inhibits endothelial proliferation and vascular morphogenesis [20]. The importance of Spry2 and Spry4 to vascular development was also shown in loss-of-function studies where both genes were deleted [21]. Loss of *Spry1* leads to abnormal kidney development and is neonatal lethal [39]. In this report, we did not observe a dramatic effect of Spry1 on endothelial cell development by gain- and loss- of function of studies on E9.5 embryos, suggesting that Spry1 has little effect on endothelial cell formation. However, because Spry1, Spry2, and Spry4 are all expressed in Flk1^+^ mesodermal cells and expressed in VEC^+^ cells, other Spry proteins may compensate for the effect of changes in Spry1 expression on endothelial formation. Although endothelial cell development in *Spry1;Tie2-Cre* embryos is normal, and the number of VEC^+^ cells in whole mount stained E9.5 yolk sacs of *Spry1;Tie2-Cre* appears similar to or greater than wild type controls, there is a failure of vascular remodeling in *Spry1;Tie2* yolk sacs as evidenced by a lack of larger vessels. Vascular integrity also appears compromised in *Spry1;Tie2-Cre* yolk sacs because autofluorescent blood cells were not contained with in vessels the way they are in wild type control yolk sacs.

Hematopoietic cells derive from hemogenic endothelial cells, which express Tie2, Flk1, VEC, and endoglin all markers of endothelial cells [26] and expression of these endothelial marker genes are decreased after hematopoietic commitment and differentiation. By FACS analysis we also showed that newly emerging hematopoietic cells (cKit^+^ and CD41^+^ cells) co-express Tie2 and Flk1 both in wild type and *Spry1;Tie2-Cre* embryos and yolk sacs. It is reasonable to expect that in wild type embryos mature blood cells do not express endothelial markers, however in *Spry1;Tie2-Cre* mice, over-expression of Spry1 may delay the down-regulation of endothelial markers in committed hematopoietic cells even after further differentiation. Further study is necessary to address this phenomenon.

Although endothelial cell development seems unaffected by over-expression of Spry1, we observed vascular defects including discontinuous endocardium and failure of vascular invasion of the neural tube in *Spry1;Tie2-Cre* transgenic embryos suggesting Spry1-expressing endothelial cells have impaired functions in vivo. Because Sprys inhibit branching morphogenesis in *Drosophila* and mice [20], [40], and vascular network formation of HUVEC on Matrigel [41], [42], it is possible that the vascular defects we observed in *Spry1;Tie2-Cre* yolk sacs and embryos is due to Spry1 over expression directly, or alternatively this defect may be indirectly the result of reduced hematopoietic cells and blood flow. Other studies have shown that defects in hematopoiesis contribute to vascular remodeling defects through changes in hemodynamic forces and cytokine production [43], [44]. To gain more insight into the vascular defects associate with Spry expression, additional studies using endothelial cell specific Cre-mediated gain- and loss-of-function of *Spry1* alone or in combination with other Spry family members will be necessary to address this issue.

## Materials and Methods

### Ethics Statement

All experiments using mice were approved by the Maine Medical Center Institutional Animal care and Use Committee and conformed to the guidelines established in *The Guide for the Use and Care of Laboratory Animals*.

### Materials

Antibodies against Flk1 and VE-Cadherin were purchased from Santa Cruz Biotechnology Inc. (Santa Cruz, CA). Phospho-ERK1/2 and p-Akt antibodies were from Cell Signaling Technology (Beverly, MA). Cy3-anti-Myc was from Sigma-Aldrich Co. (St. Louis, MO). Phosphor-Histone 3 antibody was from Millipore (Billerica, MA). FITC-anti-PCNA was from Millipore. FITC-anti-CD41, FITC-anti-CD71, APC-CD71, Alexa Fluor 488-anti-VEC, PE-anti-Tie2, PE-anti-Flk1, APC-Flk1, PE-anti-Ter119, APC-anti-c-Kit and APC-anti-CD31 were purchased from BD Biosciences (San Jose, CA). Alexa Fluor 488-anti-goat IgG was from Invitrogen Corporation (Carlsbad, CA). Fluorescein-conjugated anti-rabbit IgG, Fluorescein-anti-Mouse IgG were from Vector Laboratories Inc. (Burlingame, CA). In Situ Cell Death Detection kit TMR Red was purchased from Roche (Indianapolis, IN). ProtoScript M-MuLV First Strand cDNA Synthesis kit was purchased from New England Bio Labs (Ipswich, MA). RT^2^ Real-Time™ SYBR Green/Fluorescein PCR Master Mix was purchased from SABiosciences (Gaithersburg, MD). Methocult GF medium was purchased from Stem Cell Technologies (Vancouver, BC, Canada).

### Transgenic Mice

*Spry1* gene targeted mice on an FVB background were from the Mouse Mutant Regional Resource Center (UC, Davis). *CAGGFP-Spry1* transgenic mice on an FVB background were generated as described previously [31]. *Rosa26LacZ;Spry1* double transgenic mice were obtained by breeding *CAGGFP-Spry1* and *Rosa26LacZ* mice (Jackson Laboratory). For evaluating the recombination *Rosa26LacZ* or *Rosa26LacZ;Spry1* female mice were bred with *Tie2-Cre* male mice (Jackson Laboratory, B6.Cg-Tg(Tek-Cre)12Flv) [27]. *CAGGFP-Spry1* female mice were bred with *Tie2-Cre* male mice to generate conditional transgenic mice. Embryos were obtained from timed matings where first observation of the vaginal plug was defined as E0.5. Mouse genotypes were confirmed by PCR using genomic DNA extracted from tails or yolk sacs. Wild type C57BL6 mice were used for Spry expression pattern profile analysis. The Maine Medical Center Research Institute Animal Care and Use Committee approved all procedures.

### ES Cell culture

Mouse ES cells were maintained on mouse MEF feeder cells in the presence of leukemia inhibitory factor (LIF) in DMEM containing 15% Knockout™ Serum replacement (Invitrogen). Embroid body (EB) formation was performed using the hanging drop method without LIF.

### Fluorescence activated cell-sorting (FACS) analysis and cell sorting

Embryos and yolk sacs from timed matings were digested in 0.5 ml 0.25% trypsin for 30 min at 37°C, digestion was stopped by addition of 1 ml 10% FBS-DMEM and separated into a single cell suspension through a 26-gauge needle. Cells were pelleted by centrifugation at 1200 rpm for 5 min. Cell pellets were resuspended into 100 ul of 0.1% BSA-PBS buffer, blocked with normal rat IgG (10 µg/ml) for 5 min at 4°C followed incubation for 30 min with fluorescent antibodies: PE-anti-Flk1, APC-anti-CD31, FITC-anti-CD41, APC-anti-CD71, PE-anti-Ter119, Alex Flor488-anti-CD144, PE-anti-Tie2. Co-stained cells were washed with 0.1% BSA-PBS buffer, and filtered with MACS Pre-Separation Filters (Miltenyi Biotec). Cells were analyzed on a BD FACSCalibur™. Antibody labeled wild type cells were sorted for Flk1^+^, CD41^+^, CD71^+^, Ter119^+^, c-Kit^+^ and Flk1^+^CD41^+^ cell populations on a BD FACSAria™. Student's *t*-test was used for statistical analysis.

### RT-PCR and Quantitative real-time PCR

Total RNA was extracted from yolk sacs or embryos from timed matings using RNeasy plus (Qiagen), or from sorted cell populations using Micro RNeasy Plus (Qiagen). The purity and concentration of RNA were measured with NanoDrop Spectrophotometer (NanoDrop Technologies) at 260 nm/280 nm. The ratios of 260 nm/280 nm of all samples were between 1.8 and 2.0. ProtoScript M-MuLV First Strand cDNA Synthesis kit (New England Biolabs) was used to generate cDNA. Quantitative real-time PCR (qPCR) of target genes was performed using SYBR Green (SABioscience) according to manufacture's instructions on an IQ5 Multicolor Real-Time PCR Detection System (BioRad). GAPDH was used as an internal reference in each reaction. Melting curve analyses using the program run in the step acquisition mode was used to verify the presence of a single amplification production. Primers for qPCR are shown in supplementary Table S1. Student's *t*-test was used for statistical analysis.

### Colony forming assays

Embryos at E9.5 were dissected out and the aorta-gonad-mesonephros (AGMs) was isolated. Yolk sacs and AGMs were digested with 0.25% trypsin for 20 min at 37°C, and passed through a 26-gauge needle to obtain single cell suspensions. Cells were pelleted by centrifugation at 1200 rpm for 5 min at 4°C. Cell pellets were resuspended in 200 µl 10% FBS-DMEM and counted. AGM cells or yolk sac cells were mixed individually with 3 ml Methocult GF medium (Stem Cell Technologies) and plated into two 3.5 cm dishes and cultured at 37°C. Colonies were counted after 7 days based on morphology. At least 6 pairs of *Spry1;Tie2-Cre* and wild type controls were used for analysis. Student's *t*-test was used for statistical analysis.

### Histology and immunostaining

Embryos and yolk sacs were collected from timed pregnant females after euthanasia according to IACUC protocol, fixed in 4% paraformaldehyde (PFA) for 2 hrs, washed with PBS and saturated in sequential 15% and 30% sucrose solutions for 3 hours each, and embedded in OCT. Embryos and yolk sacs were sectioned at 6 µM. For whole mount X-gal staining, embryos were fixed in 4% PFA for 15 minutes, washed with PBS twice and stained in Ferri-F X-gal buffer. Co-immunostaining was performed with two or more antibodies including anti-phospho-ERK, anti-VEC, FITC-anti-CD41, FITC-anti-CD71, anti-pAkt, Cy3-anti-Myc as indicated in the figure legends. Proliferating cells were detected by FITC-anti-PCNA or anti-phosphor-Histone 3 immunostaining. Apoptosis was determined by TUNEL labeling using the In Situ Cell Death Detection kit according to the manufacturer's instructions. Nuclei were visualized with either TO-PRO (Invitrogen), or 4′,6-diamidino-2-phenylindole (DAPI), and sections were mounted using fluorescent mounting media. Images were taken using a Zeiss Axioskop2 Plus fluorescence microscope. For quantification of proliferation and apoptotic cells, at least 4 pairs of embryos or yolks of *Spry1;Tie2-Cre* and wild type controls were sectioned and stained. At least 5 sections from each sample were counted for quantification.

### Data analysis

Results are presented as means ± SEM. Statistical analysis of differences between two groups was performed using the Student's *t* test. Statistical significance was determined at a value of *P*<0.05.

## Supporting Information

Figure S1 **Validation of Spry1 transgene expression by RT-qPCR.** Total RNA was extracted from E8.5 or E9.5 wild type control or Spry1;Tie2-Cre embryos. RT-qPCR was performed using mouse Spry1 and GAPDH primers. The relative level was calculated by normalizing to GAPDH.
(DOC)

Figure S2 **Failure of large vessel formation and maturation in Spry1:Tie2-Cre yolk sacs.** Whole mount staining of E9.5 yolk sacs showed over-expression of Spry1 in Tie-2 expressing cells impaired yolk sac vascular remodeling and large vessel formation. Yolk sacs from E9.5 wild type or Spry1;Tie2-Cre mice were fixed in 4% PFA for 2 hours and immunostained with VEC antibodies (green), autofluoresecent blood cells are indicated in red. The nucleus was visualized with TO-PRO-3 iodide and imaged using confocal microscopy. These images are representative of three independent experiments.
(DOC)

Figure S3 **Failure of endothelial cell invasion into the neural tube of Spry1;Tie2-Cre embryos.** E9.5 embryos were fixed in 4% PFA and sectioned at 5∼7 µM. Sections were stained with anti-Flk1 antibodies, followed by FITC-anti-rabbit antibodies. Nuclei were visualized with DAPI. Red arrows indicate Flk1^+^ cells in the neural tube of WT embryos. (nt = neural tube, cv = cardinal vein).
(DOC)

Figure S4 **Over-expression of Spry1 in Tie2-expressing cells impairs formation of hematopoietic cells.** (A) RT-qPCR of E9.5 *Spry1;Tie2-Cre* yolk sacs showed over-expression of Spry1 decreased both hematopoietic and endothelial marker gene expression. (B) Representative FACS analysis of E9.5 yolk sacs to show over-expression of Spry1 impaired primitive erythropoiesis. (C) Quantification of FACS analysis on E9.5 yolk sacs showed that over-expression of Spry1 decreased CD71^+^, Ter119^+^ erythroid and erythrocyte cells but not CD31^+^ endothelial cells. (D) Quantification of FACS assay on E8.5 embryo and yolk sac cells showed a decreased CD41^+^ hematopoietic progenitor cells but not Flk1^+^ and c-Kit^+^ cells. (E) Representative of FACS analysis to show over-expression of Spry1 decreases CD41^+^ cells but not Flk1^+^ and c-Kit^+^ cells at E9.5. Analyses were performed on E9.5 *Spry1;Tie2-Cre* and control embryos. (F) Representative of FACS analysis to show over-expression of Spry1 decreases CD41^+^ but not Flk1^+^ and c-Kit^+^ cells at E8.5.
(DOC)

Figure S5 **Hematopoietic cells originate from Tie2-expressing endothelial cells.** Pooled normal E8.5 embryo and yolk sac cells were co-stained with PE-anti-Tie2, APC-anti-c-Kit and FITC-anti-CD41 antibodies. FACS analysis showed that c-Kit+ hematopoietic progenitors and CD41+ cells co-express Tie2. Data are representative of three experiments.
(DOC)

Table S1 RT-qPCR Primers.
(DOC)
