# Correction: Spry1 Is Expressed in Hemangioblasts and Negatively Regulates Primitive Hematopoiesis and Endothelial Cell Function

**DOI:** 10.1371/annotation/eb3485a7-612b-4244-8d2a-43a80cdd15eb

**Published:** 2012-01-27

**Authors:** Xuehui Yang, Yan Gong, Robert Friesel

Funding sources were omitted from the funding statement. The correct funding statement is: "This work was supported by NIH grants R01 HL65301 and P20 RR15555 to RF. Part of this work was supported by the Progenitor Cell Analysis Core, Bioinformatics and Genomics Core supported by NIH grant P20RR018789 (D. Wojchowski, PI) and the Histopathology Core supported P20RR018789 and P30RR030927 (R. Friesel, PI). The funders had no role in study design, data collection and analysis, decision to publish, or preparation of the manuscript." 

