# Supplementary figures and images for: Spry1 Is Expressed in Hemangioblasts and Negatively Regulates Primitive Hematopoiesis and Endothelial Cell Function
Source: PLoS One. 2011 Apr 1;6(4):e18374. doi: 10.1371/journal.pone.0018374 (PMC3069969; doi:10.1371/journal.pone.0018374)

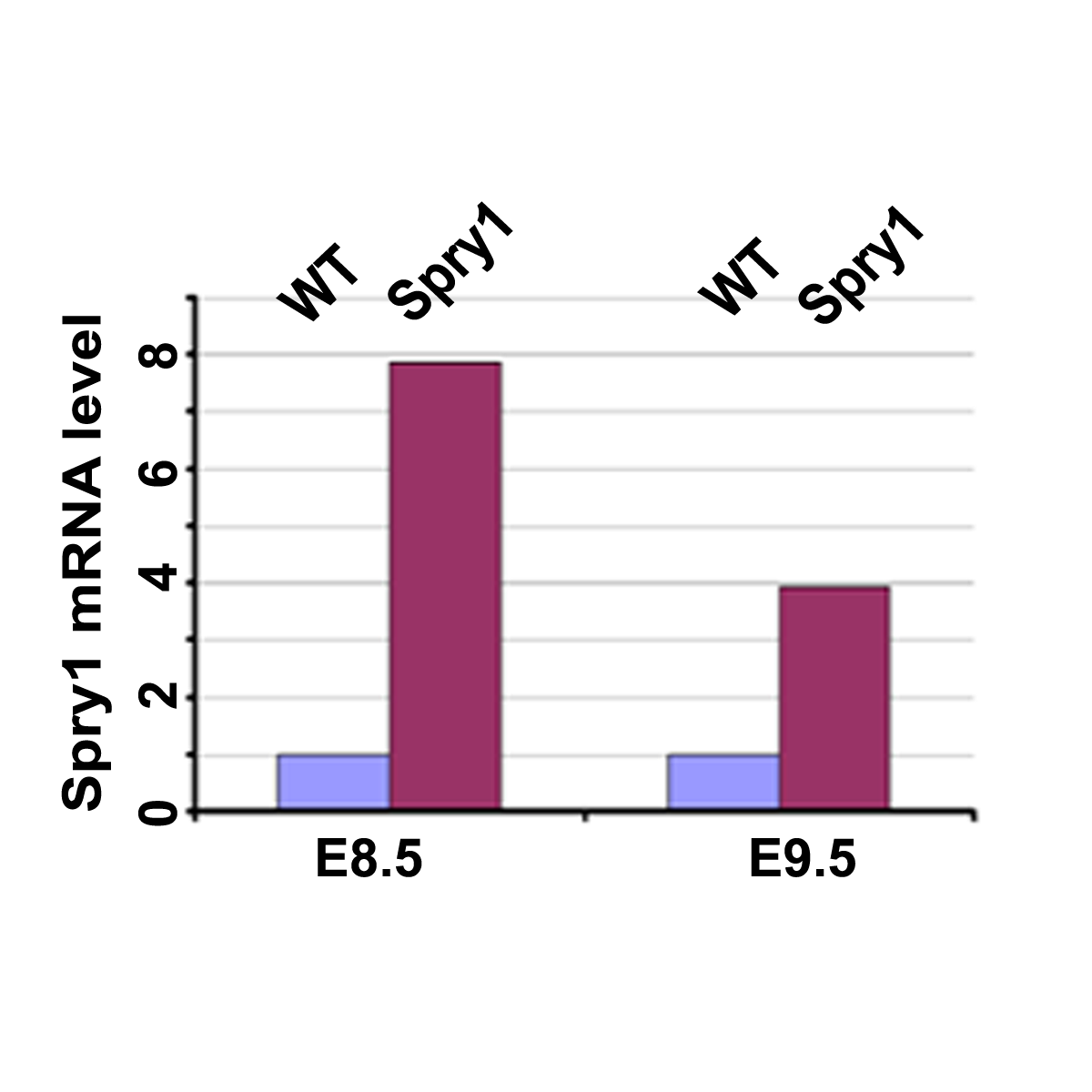

Supplement: Figure S1 — Validation of Spry1 transgene expression by RT-qPCR. Total RNA was extracted from E8.5 or E9.5 wild type control or Spry1;Tie2-Cre embryos. RT-qPCR was performed using mouse Spry1 and GAPDH primers. The relative level was calculated by normalizing to GAPDH. (DOC) [file pone.0018374.s001.doc]

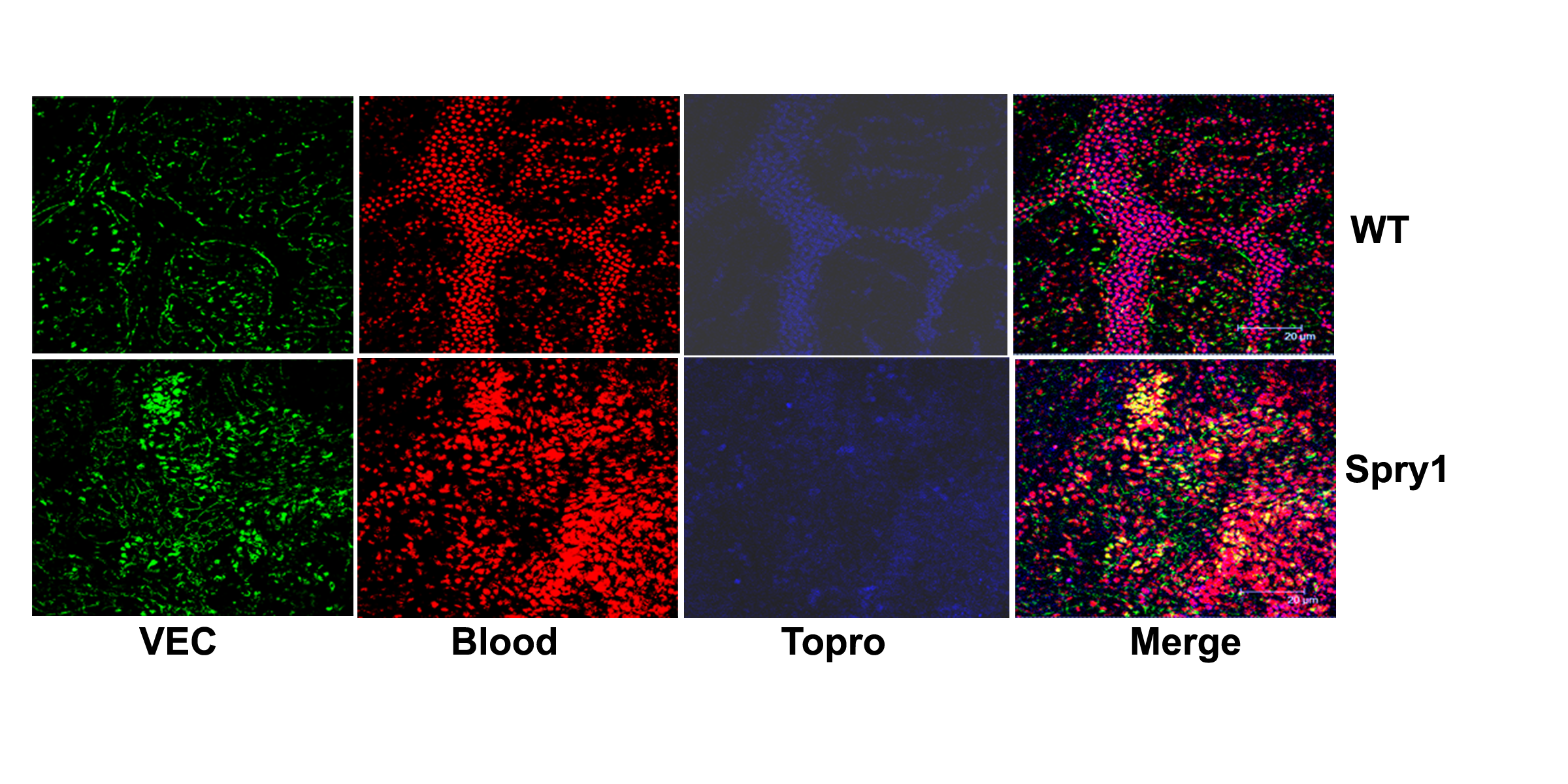

Supplement: Figure S2 — Failure of large vessel formation and maturation in Spry1:Tie2-Cre yolk sacs. Whole mount staining of E9.5 yolk sacs showed over-expression of Spry1 in Tie-2 expressing cells impaired yolk sac vascular remodeling and large vessel formation. Yolk sacs from E9.5 wild type or Spry1;Tie2-Cre mice were fixed in 4% PFA for 2 hours and immunostained with VEC antibodies (green), autofluoresecent blood cells are indicated in red. The nucleus was visualized with TO-PRO-3 iodide and imaged using confocal microscopy. These images are representative of three independent experiments. (DOC) [file pone.0018374.s002.doc]

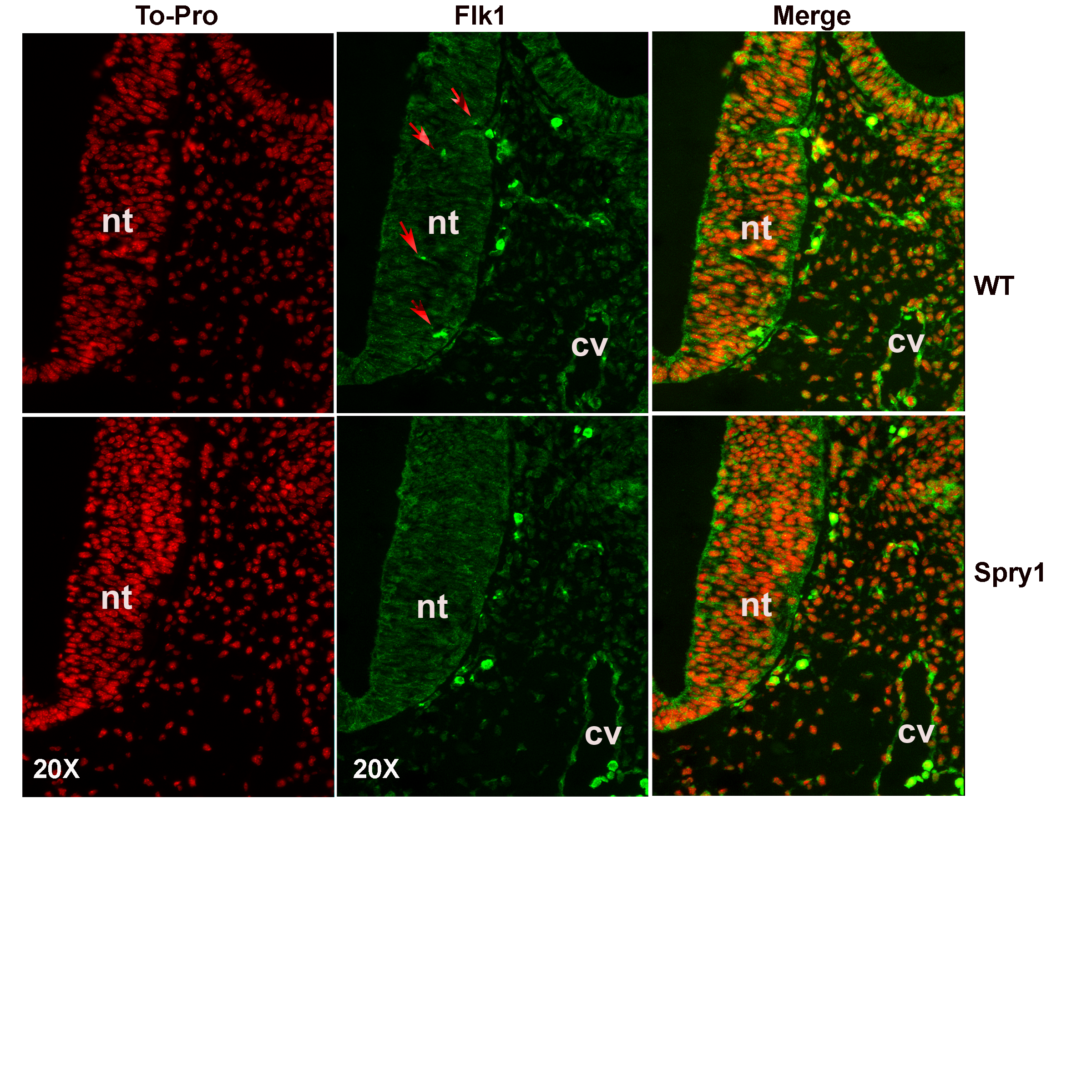

Supplement: Figure S3 — Failure of endothelial cell invasion into the neural tube of Spry1;Tie2-Cre embryos. E9.5 embryos were fixed in 4% PFA and sectioned at 5∼7 µM. Sections were stained with anti-Flk1 antibodies, followed by FITC-anti-rabbit antibodies. Nuclei were visualized with DAPI. Red arrows indicate Flk1+ cells in the neural tube of WT embryos. (nt = neural tube, cv = cardinal vein). (DOC) [file pone.0018374.s003.doc]

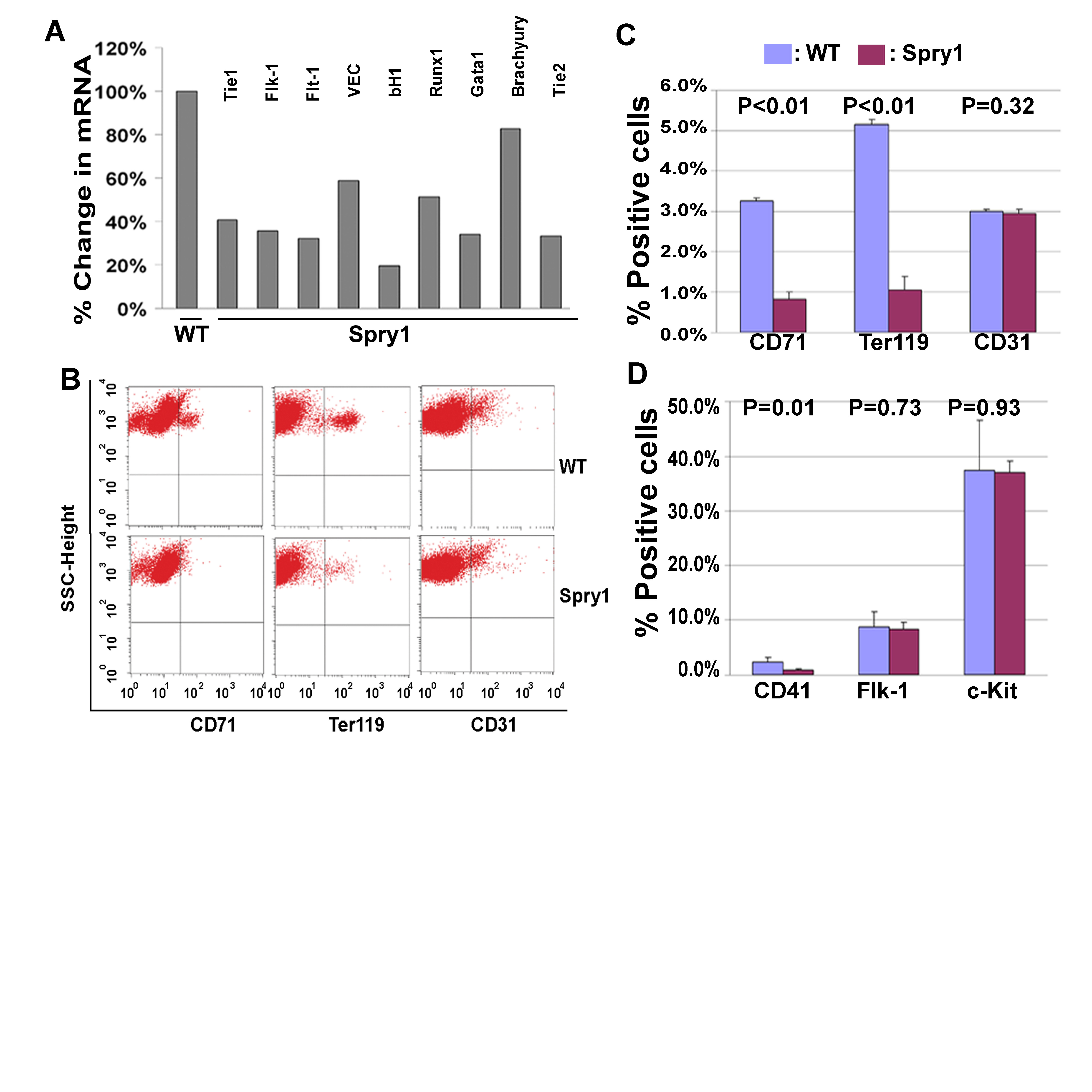

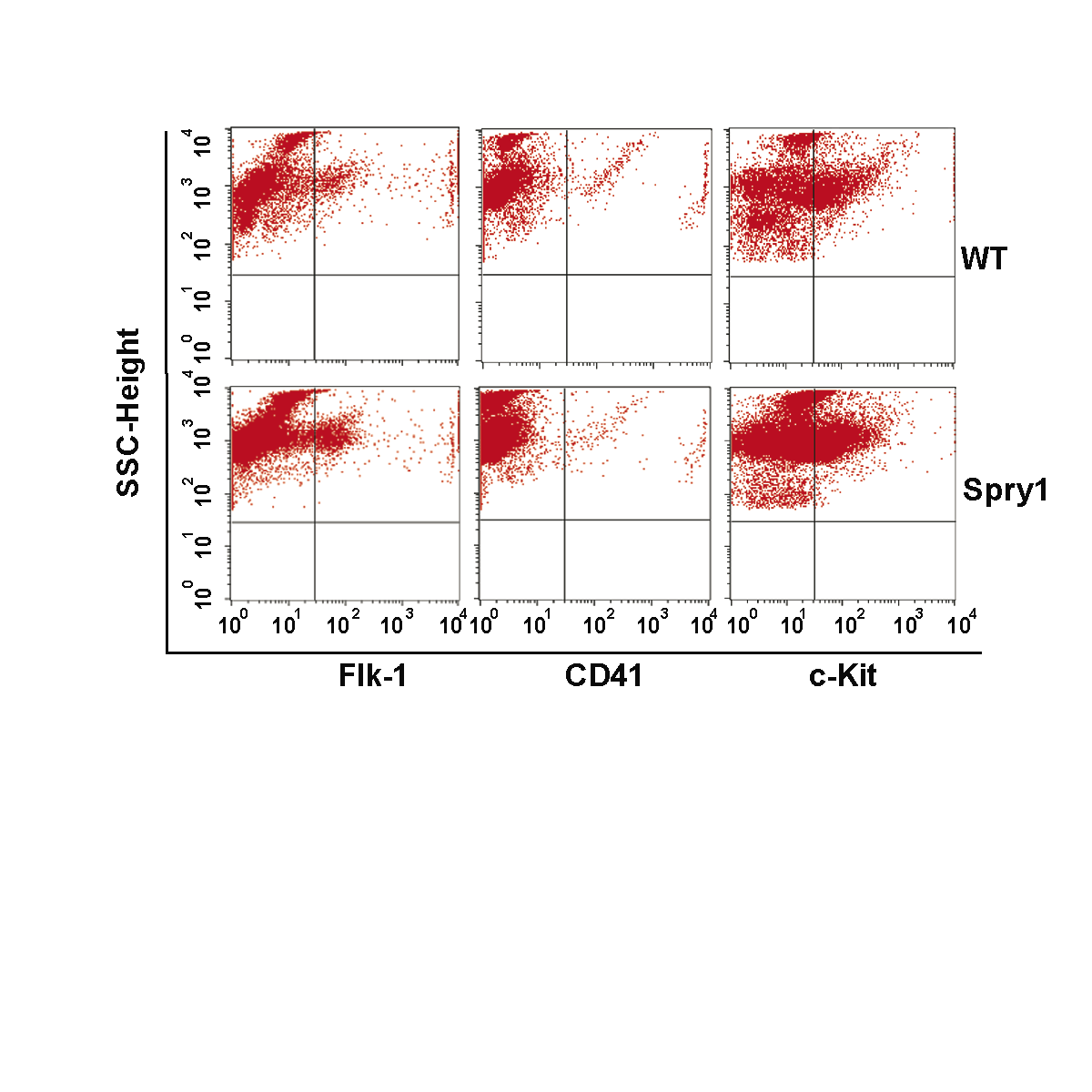

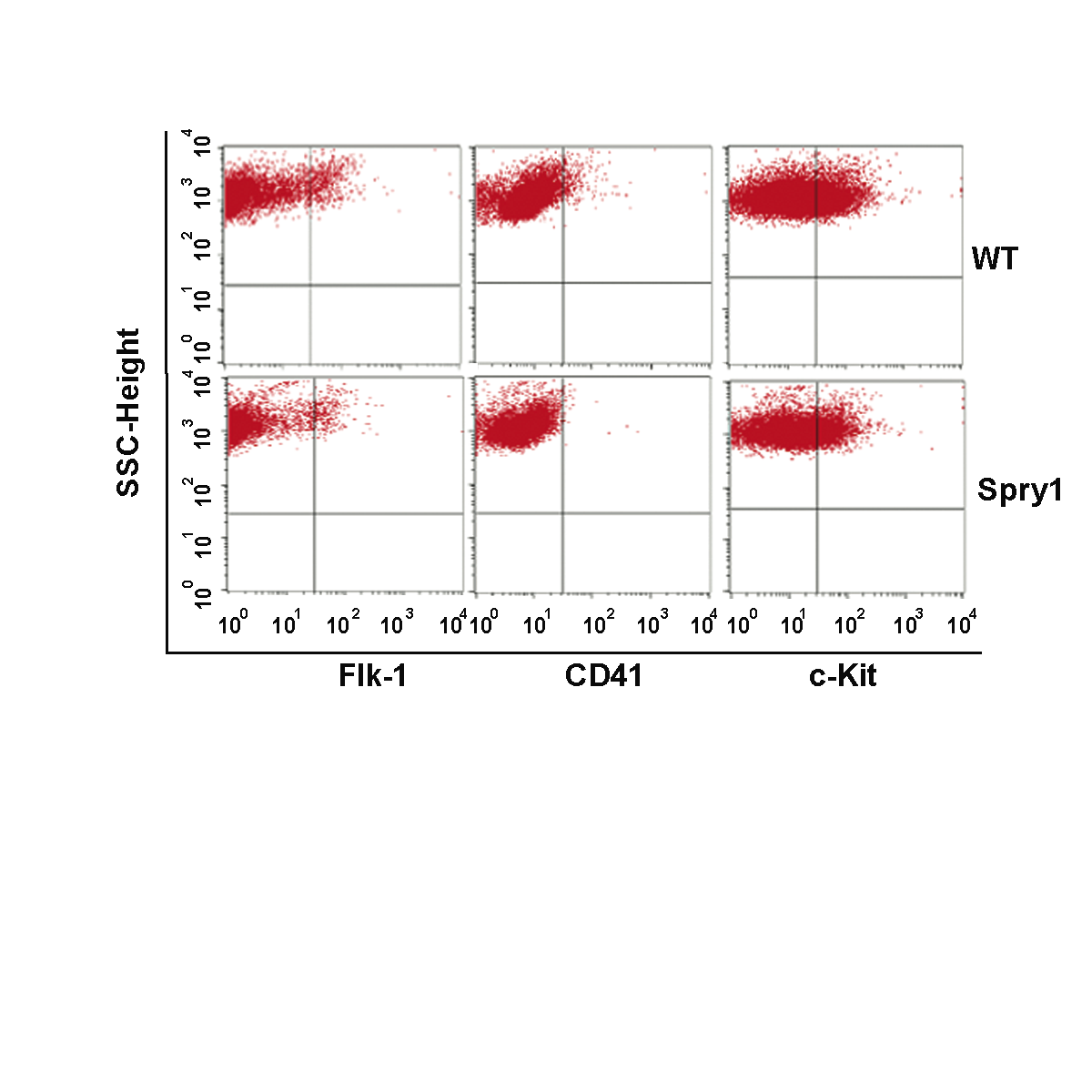


**E**

**F**

Supplement: Figure S4 — Over-expression of Spry1 in Tie2-expressing cells impairs formation of hematopoietic cells. (A) RT-qPCR of E9.5 Spry1;Tie2-Cre yolk sacs showed over-expression of Spry1 decreased both hematopoietic and endothelial marker gene expression. (B) Representative FACS analysis of E9.5 yolk sacs to show over-expression of Spry1 impaired primitive erythropoiesis. (C) Quantification of FACS analysis on E9.5 yolk sacs showed that over-expression of Spry1 decreased CD71+, Ter119+ erythroid and erythrocyte cells but not CD31+ endothelial cells. (D) Quantification of FACS assay on E8.5 embryo and yolk sac cells showed a decreased CD41+ hematopoietic progenitor cells but not Flk1+ and c-Kit+ cells. (E) Representative of FACS analysis to show over-expression of Spry1 decreases CD41+ cells but not Flk1+ and c-Kit+ cells at E9.5. Analyses were performed on E9.5 Spry1;Tie2-Cre and control embryos. (F) Representative of FACS analysis to show over-expression of Spry1 decreases CD41+ but not Flk1+ and c-Kit+ cells at E8.5. (DOC) [file pone.0018374.s004.doc]

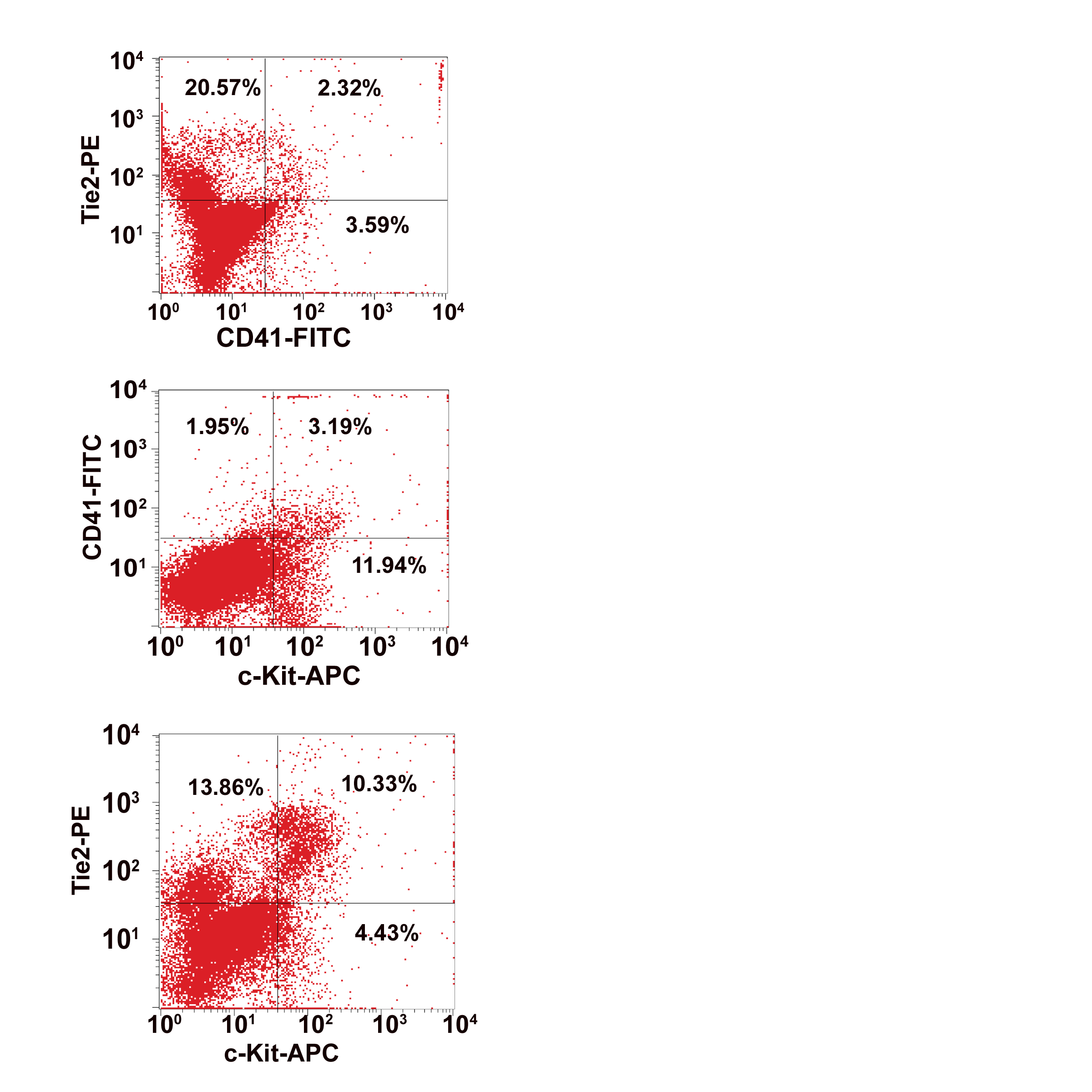

Supplement: Figure S5 — Hematopoietic cells originate from Tie2-expressing endothelial cells. Pooled normal E8.5 embryo and yolk sac cells were co-stained with PE-anti-Tie2, APC-anti-c-Kit and FITC-anti-CD41 antibodies. FACS analysis showed that c-Kit+ hematopoietic progenitors and CD41+ cells co-express Tie2. Data are representative of three experiments. (DOC) [file pone.0018374.s005.doc]
