# Supplementary material for: Spry1 Is Expressed in Hemangioblasts and Negatively Regulates Primitive Hematopoiesis and Endothelial Cell Function
Source: PLoS One. 2011 Apr 1;6(4):e18374. doi: 10.1371/journal.pone.0018374 (PMC3069969; doi:10.1371/journal.pone.0018374)
Supplement: Table S1 — RT-qPCR Primers. (DOC) [file pone.0018374.s006.doc]

| Gene name | Sequence | Gene bank  number |
| --- | --- | --- |
| Brachyury | Sense: 5’-TAAGGAACCACCGGTCATCG-3’  Antisense: 5’-TTGTCCGCATAGGTTGGAGAG-3’ | NM_009309.2 |
| Runx1 | Sense: 5’-CAACTTCCTCTGCTCCGTGC-3’  Antisense: 5’-AAAGCGATGGGCAGGGTC-3’ | NM_009821.1 |
| Gata1 | Sense: 5’-TGGGCCAGAGGGTTTGG-3’  Antisense: 5’-GCGGCCGTGGCTGCA-3’ | NM_008089.1 |
| ß-H1 | Sense: 5’-TGGGAAACCCCCGGATTA-3’  Antisense: 5’-AACCCCCAAGCCCAAGG-3’ | NM_008219.2 |
| CD41 | Sense: 5’-TGGCATGTTTCCAACCAGC-3’  Antisense: 5’-TCCCCGGTAACCATCGAA-3’ | NM_010575.1 |
| Flt-1 | Sense: 5’-ATTATGGACCCAGATGAAGT-3'  Antisense: 5'-TCACAGCCACAGTCCGGCAG-3' | NM_010228.3 |
| Flk-1 | Sense: 5'-GAGAGCAAGGCGCTGCTAGC-3'  Antisense: 5'-GACAGAGGCGATGAATGGTG-3' | NM_010612.2 |
| Tie1 | Sense: 5'-ACCCACTACCAGCTGGATGT-3'  Antisense: 5'-ATCGTGTGCTAGCATTGAGG-3' | NM_011587.2 |
| Spry1 | Sense: 5'-GTGCATGGGAGCCCTGTCTTTGTG-3’  Antisense: 5'-GTGCATGGGAGCCCTGTCTTTGTG-3’ | NM_011896.1 |
| Spry4 | Sense: 5'-CGACCAGAGGCTCCTAGATCA-3'  Antisense: 5'-CAGCGGCTTACAGTGAACCA-3' | NM_011898.2 |

Supplemental Table 1: RT-qPCR Primers
